# Anorexia nervosa—the frequency of occurrence in Polish youth, the connection with stress, auto-aggressive behaviors and the abuse of psychoactive substances

**DOI:** 10.3389/fpsyg.2025.1574136

**Published:** 2025-05-27

**Authors:** Michał Górski, Renata Polaniak, Beata Całyniuk, Jagoda Garbicz-Kata, Marta Buczkowska, Joanna Fojcik, Justyna Nowak, Joanna Domagalska

**Affiliations:** ^1^Department of Chronic Diseases and Civilization-Related Hazards, Faculty of Public Health in Bytom, Medical University of Silesia in Katowice, Katowice, Poland; ^2^Department of Human Nutrition, Faculty of Public Health in Bytom, Medical University of Silesia in Katowice, Katowice, Poland; ^3^Department of Psychiatry, Faculty of Health Sciences in Katowice, Medical University of Silesia in Katowice, Katowice, Poland; ^4^Department of Neurology, Faculty of Health Sciences in Katowice, Medical University of Silesia in Katowice, Katowice, Poland; ^5^Department of Cardiovascular Disease Prevention, Faculty of Public Health in Bytom, Medical University of Silesia in Katowice, Katowice, Poland; ^6^Department of Environmental Health, Faculty of Public Health in Bytom, Medical University of Silesia in Katowice, Katowice, Poland

**Keywords:** eating disorders, anorexia nervosa, schoolchildren, adolescents, prevalence, prevalence of anorexia nervosa, stress

## Abstract

**Background:**

Anorexia nervosa is an eating disorder characterized by a distorted self-image, an intense desire for weight loss, anxiety, and strict dieting. The disorder primarily affects children, adolescents, and young adults. It leads to serious health consequences and increases the risk of depression, suicidal thoughts, and addiction. The causes of the disorder are multifactorial, including genetic influences, social pressures, and emotional problems. Eating disorders are now considered a serious public health problem that requires special supervision by specialists and the development of long-term measures to reduce their incidence.

**Objective:**

The objectives of the study were to determine the prevalence of anorexia nervosa among school adolescents aged 15–19; to determine the relationship between the prevalence of anorexia nervosa and the severity of school stress, the severity of family stress, the use of psychoactive substances, and the occurrence of auto-aggressive and self-destructive behavior.

**Materials and methods:**

The group of respondents consisted of individuals aged 15 to 19 years (secondary school students). The study utilized a diagnostic interview method, along with an additional questionnaire. The author’s survey questionnaire was developed based on the diagnostic criteria for anorexia nervosa in the International Classification of Diseases, 11th Revision and Diagnostic and Statistical Manual of Mental Disorders, Fifth Edition, as well as information from scientific sources regarding stressors and auto-aggressive and self-destructive behaviors. In the statistical analysis, a significance level of *α* = 0.05 was adopted.

**Results:**

The diagnostic criteria for anorexia nervosa were met by 13.3% of respondents. 37.1% of respondents admitted to engaging in self-injurious behavior, and 13.9% reported having suicidal thoughts. The greatest risk factors for developing anorexia nervosa were female gender, high maternal education, and high levels of physical activity.

**Conclusion:**

Individuals who could be diagnosed with anorexia nervosa experienced higher levels of school stress and a greater sense of family stress compared to those who did not meet the diagnostic criteria for the disorder. Auto-aggressive and self-destructive behaviors were much more common among those with a possible diagnosis of anorexia nervosa than among those who did not meet the criteria for the disorder.

## Introduction

In the world, there is a dynamic increase in the number of patients diagnosed with mental disorders and illnesses (Robinson et al., 2022). According to the World Health Organization (McGorry et al., 2022) (WHO), 10–20% of adolescents require mental health professionals, and mental disorders and illnesses are the main cause of disability among children and adolescents (McGorry et al., 2022). In literature, attention is drawn to the fact of the low availability of specialists in the field of psychiatry of children and adolescents, which can delay or even make it impossible to make the correct diagnosis and conduct an adequate therapeutic process (McGorry et al., 2022).

According to statistics, eating disorders are one of the most common mental problems in the group of adolescents and young adults. They are the third most prevalent group of diseases in this age group, more commonly occur only bronchial asthma and diabetes (Appolinario et al., 2022). At present, eating disorders are regarded as a major public health problem, which requires special supervision by specialists and the development of long-term measures aimed at reducing their incidence.

It is difficult to conduct epidemiological studies on eating disorders because of certain factors. Firstly, patients often mask the symptoms of the disease, hide the problems for a long time and ask late (or not at all) for help, which makes it nearly impossible to diagnose these disorders (Qian et al., 2022). A significant problem is also the wide range of somatic symptoms that occur, which makes the initial diagnosis usually targeted to other diseases (Galmiche et al., 2019). The difficulty is also the co-occurrence of other mental disorders, such as affective disorder, substance addiction or personality disorder (Qian et al., 2022; Galmiche et al., 2019; Kjeldbjerg and Clausen, 2023). The stigmatisation of mental illness is also a widely debated phenomenon, which is becoming a hindering factor in the conduct of epidemiological research in the field of mental health (O’Connor et al., 2021). Patients often feel rejected because of symptoms, deny the occurrence of the disease (especially in the case of anorexia nervosa and bigorexia) and feel ashamed and overwhelmed (particularly in bulimia nervosa and compulsive eating) (O’Connor et al., 2021). Another factor that prevents correct diagnosis is the lack of awareness and selective knowledge about eating disorders among health workers. Inadequate competence in this area often causes patients to undergo many visits and consultations with different specialists before a final diagnosis is made (Johns et al., 2019).

Anorexia nervosa is a disorder characterised by significantly low body weight relative to height, age, and the individual’s biopsychophysical developmental stage (Mehler et al., 2022). Importantly, this condition is not caused by another medical illness that impairs food intake or absorption, nor is it the result of food unavailability (Broomfield et al., 2021). A core symptom of the disorder is marked anxiety and intense fear of weight gain, accompanied by a distorted body image, typically involving an exaggerated perception of body size and an atypical perception of body shape (Pettersson et al., 2021).

Affected individuals alter their eating habits, adopt excessively restrictive energy intake regimens, and deliberately limit food consumption, which leads to weight loss and, ultimately, to physical emaciation and potentially death (Mehler et al., 2022; Pettersson et al., 2021).

According to the International Classification of Diseases, 11th Revision (ICD-11) diagnostic criteria (World Health Organization, 2021), anorexia nervosa can be diagnosed when the following conditions are met:

- Significantly low body weight relative to the individual’s height, age, and developmental stage, not attributable to another disease or lack of access to food;
- A body mass index (BMI) below 18.5 kg/m^2^ in adults, or below the 5th percentile BMI-for-age in children and adolescents;
- Low body weight is accompanied by persistent behaviors that prevent weight restoration (e.g., restrictive eating, purging behaviors, or behaviors that increase energy expenditure);
- An intense fear of gaining weight;
- Self-esteem is strongly influenced by maintaining a low body weight and a slim physique (with underweight perceived as normative or even ideal).

- Rapid weight loss over a short period may substitute for the criterion of persistently low body weight, provided that the other diagnostic criteria are fulfilled.
- In children and adolescents, failure to gain weight according to their growth trajectory may be considered a diagnostic criterion (instead of weight loss).

The progression of the illness, especially in its early stages, is often difficult to detect, as individuals tend to conceal symptoms—for example, by hiding weight loss under loose clothing or avoiding social situations involving meals (Broomfield et al., 2021). As a result, anorexia nervosa is often diagnosed at a later stage (Broomfield et al., 2021).

The disorder is typically chronic, manifesting either as a single, prolonged episode or as recurring periods of remission and relapse. Numerous factors influence the severity and treatment outcomes, with the most significant being early diagnosis, prompt treatment initiation, a supportive environment, and strong motivation to recover (Mehler et al., 2022; Broomfield et al., 2021; Pettersson et al., 2021).

The main objective of the study was to determine the incidence of anorexia nervosa among schoolchildren aged 15–19 years. Furthermore, the specific objectives were considered to determine the relationship between the incidence of anorexia nervosa in the study group and:

- The intensification of school stress
- The exacerbation of family stress
- Use/abuse of psychoactive substances
- The occurrence of auto-aggressive and self-destructive behavior.

Before conducting the study, the following main hypothesis was formulated: the prevalence of anorexia nervosa in Polish youth is significantly associated with psychological stress, auto-aggressive behaviors, and psychoactive substance abuse.

The following specific hypotheses were also formulated:

- Higher levels of perceived stress are correlated with a greater risk of developing anorexia nervosa among Polish adolescents.
- Youth diagnosed with anorexia nervosa report a higher incidence of auto-aggressive behaviors compared to their peers without the disorder.
- There is a statistically significant relationship between anorexia nervosa and the use of psychoactive substances in Polish adolescents.

## Method

The group of respondents consisted of individuals aged 15 to 19 years (secondary school students). This age group was included in the study because adolescence is associated with a high risk of developing anorexia nervosa. It is a period marked by intense biological, psychological, and social changes that may contribute to body image disturbances and the emergence of disordered eating patterns. The study was conducted between September 2020 and December 2024. A total of 1900 questionnaires were distributed, and data from 1,696 students (89% of the issued surveys) were analyzed. In the process of data analysis, 77 surveys (4% of all surveys) were excluded due to incorrect completion, such as selecting multiple answers for single-choice questions or submitting an unfilled survey. Additionally, 125 surveys (7%) were not returned for evaluation after distribution.

Respondents were selected using a multi-stage random sampling method. Participation in the study was voluntary. The research project was approved by the Head of the Department of Education and the headmasters of the schools involved in the study. According to Polish law, this study did not qualify as a medical experiment and therefore did not require approval from the Bioethics Committee (Act of 5 December 1996 on the professions of physician and dentist [Journal of Laws of 2019, para. 537]).

The main inclusion criteria for participation in the study were:

- Age (15–19 years),
- Consent to participate in the survey,
- Maintenance of basic cognitive competencies necessary for completing the survey questionnaire and answers to the interview questions (such as reading, writing, and understanding).

Exclusion criteria included:

- A diagnosis of mild intellectual disability,
- Enrollment in special schools or life-adapting schools (while students with decisions regarding the need for special education were included, the completion of the survey required assistance from a teacher or guardian).

In addition to the survey, the study also included an individual medical interview with each child. The interview was conducted in a private, confidential setting, ensuring that the respondent felt comfortable and secure during the process. At the request of the respondent, any family member, guardian, or trusted person could be present during the interview to provide support.

The medical interview was conducted by a specialized medical team, ensuring that each interview was handled with the utmost professionalism and care. The medical team was composed of specialists working with children and adolescents, including physicians (such as psychiatrists and pediatricians), psychologists, dietitians, and nurses. All respondents answered the same set of questions, which were focused on topics related to mental health, stress levels, family dynamics, and behavior patterns associated with anorexia nervosa. The questions were designed to assess not only the psychological and emotional aspects of the respondents’ experiences but also their physical health, eating habits, and any signs of self-destructive behavior or auto-aggression.

The survey questionnaire consisted of two parts. The first part collected metric data. Based on body weight and height, the body mass index (BMI) was calculated and interpreted according to the BMI centile grids for the Polish population of children and adolescents from Ola and Olaf (Kułaga et al., 2015) (adolescents up to 18 years of age) and according to the WHO guidelines (World Health Organization, 2023) for those over 18 years of age. Tables 1–3 present the metric data for the group.

**Table 1:** Basic metric data of the study group (source: own study).

Variable	X ± SD	Minimum	Maximum
Age [years]	16.9 ± 2.44	15	19
Weight [kg]	62.7 ± 13.31	34.0	142.0
Height [cm]	170.3 ± 9.28	135	202
BMI [kg/m^2^]	21.5 ± 3.53	13.1	42.8

**Table 2:** Characteristics of the group examined, including gender distribution (source: own study).

Variable	Overall *n*;%	Gender		*p* value
		Women *n*;% 1.064; 62.7%	Men *n*;% 632; 37.3%	
Age (years)				
15	349; 20.6%	211; 19.8%	138; 21.8%	0.44*
16	427; 25.2%	256; 24.1%	171; 27.1%	
17	352; 20.7%	210; 19.7%	142; 22.5%	
18	324; 19.1%	222; 20.9%	102; 16.1%	
19	244; 14.4%	165; 15.5%	79; 12.5%	
Place of residence				
Village	751; 44.3%	445; 41.8%	306; 48.4%	0.07*
Small town (up to 20 thousand inhabitants)	141; 8.3%	98; 9.2%	43; 6.8%	
Medium-sized city (between 20 thousand and 60 thousand inhabitants)	337; 19.9%	214; 20.1%	123; 19.5%	
Big city (between 60 thousand and 100 thousand inhabitants)	211;12.4%	140; 13.2%	71; 11.2%	
Very large city (100 thousand and more inhabitants)	256; 15.1%	167; 15.7%	89; 14.1%	
Type of school				
Trade school	306; 18%	113; 10.6%	193; 30.5%	<0.0001*
Technical school	754; 44.5%	441; 41.4%	313; 49.5%	
High school	636; 37.5%	510; 47.9%	126; 19.9%	
Physicala ctivity				
Verylow (<15 min daily)	323; 19%	245; 23.0%	78; 12.3%	<0.0001*
Low (15–30 min daily)	459; 27.1%	328; 30.8%	131; 20.7%	
Normal (31–60 min daily)	589; 34.7%	356; 33.5%	233; 36.9%	
High (61–120 min daily)	193; 11.4%	95; 8.9%	98; 15.5%	
Very high (>120 min daily)	132; 7.8%	40; 3.8%	92; 14.6%	
Diagnosed eating disorders				
No	1.638; 96.6%	1.014; 95.3%	624; 98.7%	0.0002**
Yes	58; 3.4%	50; 4.7%	3; 0.5%	
Presence of chronic disease				
No	1.479; 87.2%	887; 83.4%	592; 93.8%	<0.0001*
Yes	217; 12.8%	177; 16.6%	39; 6.2%	
Family financial position				
Low	20; 1.2%	12; 1.1%	8; 1.3%	0.31*
Sufficient	268; 15.8%	176; 16.5	92; 14.6%	
Good	1.061; 62.6%	648; 60.9%	413; 65.3%	
Very good	347; 20.4%	228; 21.4%	119; 18.8%	
Having siblings				
No siblings	245; 14.4%	152; 14.3%	93; 14.7%	0.07*
1 sister or 1 brother	764; 45%	479; 45.0%	285; 45.1%	
2 siblings	394; 23.3%	265; 24.9%	129; 20.4%	
3 and more siblings	293; 17.3%	168; 15.8%	125; 19.8%	
Mother’s education				
Primary	52; 3.1%	31; 2.9%	21; 3.3%	0.0029*
Vocational	401; 23.6%	254; 23.9%	147; 23.3%	
Secondary	476; 28.1%	314; 29.5%	162; 25.6%	
Higher	543; 32%	352; 33.1%	191; 30.2%	
Do not know	186; 11%	95; 8.9%	91; 14.4%	
Does not apply	38; 2.2%	18; 1.7%	20; 3.2%	
Father’s education				
Primary	55; 3.2%	29; 2.7%	26; 4.1%	0.06*
Vocational	586; 34.6%	373; 35.1%	213; 33.7%	
Secondary	434; 25.6%	287; 27.0%	147; 23.3%	
Higher	317; 18.7%	203; 19.1%	114; 18.0%	
Do not know	234; 13.8%	135; 12.7%	99; 15.7%	
Does not apply	70; 4.1%	37; 3.5%	33; 5.2%	

* test χ^2^. ** Fisher’s test.

**Table 3:** Frequency of diagnosed eating disorders and selected chronic diseases among respondents (source: own study).

Variable	Overall *n*;%	Gender		*P* value
		Women *n*;%	Men *n*;%	
Diagnosed eating disorders (based on respondents’ declarations)				
Bulimia nervosa	22; 1.3%	22; 2.1%	0; 0%	<0.0001**
Anorexia nervosa	19; 1.1%	17; 1.6%	2; 0.3%	0.016**
Compulsive overeating	7; 0.4%	6; 0.6%	1; 0.2%	0.27**
Eating disorders unspecified	7; 0.4%	4; 04%	3; 0.5%	0.71**
Orthorexia nervosa	2; 0.1%	2; 0.2%	0; 0%	0.53**
Bigorexia nervosa	1; 0.06%	1; 0.1%	0; 0%	0.44**
Chronic diseases				
Depression	75; 4.4%	62; 5.8%	13; 2.1%	0.0003*
Anxiety disorder	21; 1.2%	16; 1.5%	5. 0.6%	0.04**
Bronchial asthma	21; 1.2%	15; 1.4%	6; 0.9%	0.50**
Autoimmune chronic thyroiditis (Hashimoto’s disease)	14; 0.8%	11; 1.0%	3; 0.5%	0.28**
Diabetes	6; 0.4%	5; 0.5%	1; 0.2%	0.42**
Hyperthyroidism	6; 0.4%	5; 0.5%	1; 0.2%	0.42**
Epilepsy	5; 0.3%	5; 0.5%	0; 0%	0.16**
Bipolar affective disorder	4; 0.2%	3; 0.3%	1; 0.2%	0.98**
Coeliac disease	4; 0.2%	2; 0.2%	2; 0.3%	0.63**

* test χ^2^. ** Fisher’s test.

The second part consisted of the author’s questionnaire, which was based on the diagnostic criteria for anorexia nervosa as outlined in the International Classification of Diseases, 11th edition (ICD-11) (World Health Organization, 2021), and the Diagnostic and Statistical Manual of Mental Disorders, 5th edition (DSM-5) (American Psychiatric Association, 2013).

To assess the severity of stress associated with school and family life, created a table listing possible stressors. The respondents evaluated the presence of each stressor in their lives using a six-point scale (0–5), based on the Likert scale, where 0 indicated that the stressor existed but was not a source of stress, 1 indicated minimal stress, 5 indicated maximum stress. In cases where a stressor was absent from the respondent’s life, the option “does not apply” could be selected. To assess the occurrence of auto-aggressive and self-destructive behaviors, the questionnaire included questions about deliberate (non-accidental) injury to one’s body and suicidal behavior.

Participation in the study was voluntary. Prior to conducting the study, the survey was validated. The validation process involved 80 participants, who were asked to fill out the questionnaire twice at a twenty-one-day interval. The results were statistically analyzed to assess the reliability of the questionnaire. Internal consistency was tested using Cronbach’s alpha coefficient, and the correlation coefficients between the responses to individual questions and the total scale scores were determined. Test–retest reliability was assessed by comparing the results obtained from the same individuals completing the questionnaire twice, with a 21-day interval, and calculating the intraclass correlation coefficient (ICC).

A significantly high correlation was found between the scores for each question and the total score (*p* < 0.05, r > 0.69). The Cronbach’s alpha coefficient was calculated to be 0.88, indicating very good internal consistency for the questionnaire. Reliability analysis was based on correctly completed questionnaires filled out twice. The level of repeatability was assessed using the ICC coefficient, which was 0.82. No statistically significant differences were found between the total scores or individual question scores obtained after completing the questionnaire twice (on day 0 and day 21) (*p* > 0.05 in each case). Correlation coefficients between answers to individual questions from the first and second completions of the questionnaire showed a significant and high correlation (*p* < 0.05, r > 0.55 for each question).

Statistical analysis was performed using the programs STATISTICA 13.3 PL (StatSoftPolska, Krakow, Poland) and SAS 9.4 (SAS Institute Inc., Cary, NC, United States). Quantitative data were presented as mean values (X) along with standard deviation (SD). The frequency of individual responses indicated by the respondents was described using the number “n” and as a percentage of the group’s responses. The normality of distributions was assessed using the Shapiro–Wilk test. Homogeneity of variances was evaluated with Levene’s test. To determine the statistical significance of qualitative variables, the chi-square test or Fisher’s exact test was used.

To identify variables that constituted significant risk factors, univariate logistic regression analyses were conducted, which served as the basis for developing multivariate models. In the multivariate analyses, variables from the univariate analyses with a *p*-value < 0.3 were selected for the initial model. In the analysis of school and family stress intensity, the average number of points was considered rather than individual question scores. Next, an automatic stepwise elimination procedure was performed in the multivariate model, with an entry criterion of *p* < 0.2 and a retention criterion of *p* < 0.3. Ultimately, only those variables with a *p*-value < 0.05 were considered significant risk factors. Variables meeting this criterion remained in the model and were recognized as independent risk factors for a given eating disorder.

A significance level of *α* = 0.05 was adopted for statistical analysis.

## Results

### Anorexia nervosa—diagnostic criteria and picture of the disease

10.2% of the total subjects (*n* = 173) met the diagnostic criterion BMI below 18.5 kg/m^2^ or below 5 percentiles. BMI below the 5th percentile was significantly more common in the female group (13.1%) than in the male group (5.4%) (*p* < 0.001). Up to 43% of respondents admitted that they felt anxiety about weight gain. Indeed, women were more likely to suffer from this anxiety (59.3%) than men (15.5%). One in four respondents avoided foods that in society are considered to be “fattening,” e.g., butter or chocolate. Women were more likely to avoid this type of food (*n* = 327; 30.7%) than men (*n* = 106; 16.8%). Nearly one in five respondents (*n* = 324; 19.1%) admitted to using compensatory methods. 71.9% of all respondents (*n* = 1.220) regarded appearance and body shape as one of the most important aspects of life and over-focused on body weight. This was indicated by the vast majority of women (*n* = 850; 79.9%) and by more than half of men (*n* = 370; 58.8%).

A similar number of respondents in general (*n* = 1.213; 71.5%) denied excessive interest in the energy value of food products. Excessive concentration in this aspect was indicated by 16.1% of men (*n* = 102) and 35.8% of women (*n* = 381). Nearly half of the respondents (*n* = 843; 49.7%) thought that their body was unattractive, despite people around them having a different opinion on the subject. This belief was characterized by 62.9% of women (*n* = 669) and 27.5% of men (*n* = 174). Less than two-thirds of respondents (64.4%) admitted that they compared the appearance and shape of their bodies with that of their peers. This response was significantly more common among women (*n* = 827; 77.7%) than among men (42.1%, *n* = 266). A significant number of respondents (40.9%) admitted that body weight and body shape were the cause of unpleasant comments from the environment, where such remarks experienced as much as 47.3% of women and 30.2% of men. One in three respondents divided their meals into very small portions to make them look bigger. 43.6% of women and 21.2% of men reflected such behavior. The vast majority of respondents (85.8%, *n* = 1.456) denied counting food bits.

Nearly one in three subjects (*n* = 539; 31.8%) admitted that they weigh more than 3 times a week, with significantly more women (*n* = 420; 39.5%) than men (*n* = 119; 18.8%) (*p* < 0, 0001). Nearly one in five subjects (*n* = 318; 18.8%) wore too large clothes to hide weight loss from their surroundings, but women (25.5%) were more likely to have such behavior than men (7.4%). The exact data are presented in Table 4.

**Table 4:** Diagnostic criteria for anorexia nervosa and selected aspects of the clinical picture of the disease, including gender distribution (source: own study).

Diagnostic criterion/picture of the disease	Yes/No	Overall *n*;%	Gender		*P* value
			Woman *n*; %	Man *n*; %	
BMI below 18.5 kg/m^2^ or below 5 percentyl	Yes	173; 10.2%	139; 13.1%	34; 5.4%	<0.001*
	No	1.523; 89.8%	925; 86.9%	598; 94.6%	
There is fear of weight gain	Yes	729; 43%	631; 59.3%	98; 15.5%	<0.0001*
	No	967; 57%	433; 40.7%	534; 84.5%	
Avoids “fatty” food. like butter. bread. chocolate	Yes	433; 25.5%	327; 30.7%	106; 16.8%	<0.0001*
	No	1.263; 74.5%	737; 69.3%	526; 83.2%	
Uses compensatory methods	Yes	324; 19.1%	238; 22.4%	86; 13.6%	<0.0001*
	No	1.372; 80.9%	826; 77.6%	546; 86.4%	
Focuses excessively on body weight. recognizing appearance and body shape as one of the most important aspects of life	Yes	1.220; 71.9%	850; 79.9%	370; 58.8%	<0.0001*
	No	476; 28.1%	214; 20.1%	262; 41.5%	
Overly interested in the energy value of food (knows the energy value of more than 10 products)	Yes	483; 28.5%	381; 35.8%	102; 16.1%	<0.0001*
	No	1.213; 71.5%	683; 64.2%	530; 83.9%	
Believes that the body is unattractive. despite the fact that people around him think differently	Yes	843; 49.7%	669; 62.9%	174; 27.5%	<0.0001*
	No	853; 50.3%	394; 37.1%	458; 72.5%	
Compares the appearance and shape of one’s body to peers’ body	Yes	1.093; 64.4%	827; 77.7%	266; 42.1%	<0.0001*
	No	603; 35.6%	237; 22.3%	366; 57.9%	
Appearance (weight and body shape) were the cause of unpleasant comments from the environment	Yes	694; 40.9%	503; 47.3%	191; 30.2%	<0.0001*
	No	1.002; 59.1%	561; 52.7%	441; 69.8%	
Divides the meal into very small parts to give the impression of a larger portion than it actually is	Yes	598; 35.3%	464; 43.6%	134; 21.2%	<0.0001*
	No	1.098; 64.7%	600; 56.4%	498; 78.8%	
Counts billets of food to eat a certain number of them	Yes	240; 14.2%	164; 15.4%	76; 12%	<0.01*
	No	1.456; 85.8%	900; 84.6%	556; 88%	
Weighs more than 3 times a week	Yes	539; 31.8%	420; 39.5%	119; 18.8%	<0.0001*
	No	1.157; 68.2%	644; 60.5%	513; 81.2%	
Deceives the environment as to the actual body weight (gives the overstated or reduced result)	Yes	480; 28.3%	401; 37.7%	79; 12.5%	<0.0001*
	No	1.216; 71.7%	663; 62.3%	553; 87.5%	
Wears too big clothes to hide his weight loss	Yes	318; 18.8%	271; 25.5%	47; 7.4%	<0.0001*
	No	1.378; 81.2%	793; 74.5%	585; 92.6%	

* test χ^2^.

**Figure 1:**
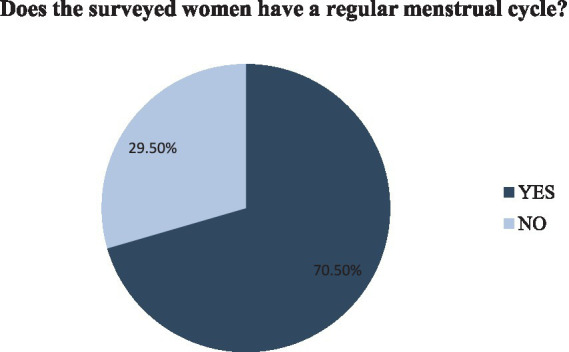
Regularity of the menstrual cycle in women surveyed (source: own study).

Nearly one in three women surveyed (*n* = 313; 29.5%;) admitted having an irregular menstrual cycle. This problem did not affect 70.5% of respondents (*n* = 751) (Figure 1).

As much as 13.3% of all respondents (*n* = 226) met the diagnostic criteria for anorexia nervosa based on ICD-11, with this disease being significantly more prevalent in women than in men (accordingly, 19.7% vs. 2.5%) (Table 5).

**Table 5:** Possible diagnosis of anorexia nervosa based on the diagnostic criteria of ICD-11 (source: own study).

Diagnostic criteria for anorexia nervosa	Category	Overall *n*;%	Gender		*P* value
			Woman *n*; %	Man *n*; %	
Meeting the diagnostic criteria for anorexia nervosa	Yes	226; 13.3%	210; 19.7%	16; 2.5%	<0.0001*
	No	1.470; 86.7%	854; 80.3%	616; 97.5%	

* test χ^2^.

Among the subjects with a possible diagnosis of anorexia nervosa, 60.2% of respondents abused psychoactive substances. School stress >90 percentile was characterized by 26.1% of respondents who met the diagnostic criteria for anorexia nervosa compared to 7.0% of those who did not meet these criteria. Statistically significant results (*p* < 0.0001) were also obtained for high sense of family stress (>90 percentile), which characterized 18.6% of people with a possible diagnosis of AN and 9.6% of those without such diagnosis. As many as 72.1% of respondents who showed symptoms of anorexia nervosa had auto-aggressive behavior, 34.5% of the group admitted that they had had suicidal thoughts in the past or during the study, and 26.5% of those surveyed admitted having attempted suicide in their past (Table 6).

**Table 6:** Possible diagnosis of anorexia nervosa taking into account selected variables (source: own study).

Variable	Category	Overall *n*; %	Possible recognition AN		*P* value
			Yes *n*; %	No *n*; %	
Abuse of psychoactive substances	Yes	815; 48.1%	136; 60.2%	679; 46.2%	<0.0001*
	No	881; 51.9%	90; 39.8%	791; 53.8%	
Stress at school>90 percentyl	Yes	159; 9.4%	59; 26.1%	100; 7.0%	<0.0001*
	No	1.537; 90.6%	167; 73.9%	1.370; 93.0%	
Stress in family > 90 percentyl	Yes	170; 10.0%	41; 18.6%	129; 9.6%	<0.0001*
	No	1.526; 90.0%	180; 81.4%	1.346; 90.4%	
Auto-aggressive behavior in the past or during the study	Yes	629; 37.1%	163; 72.1%	466; 31.7%	<0.0001*
	No	1.067; 62.9%	63; 27.9%	1.004; 68.3%	
Suicidal thoughts in the past or during the study	Yes	235; 13.9%	78; 34.5%	157; 10.7%	<0.0001*
	No	1.461; 86.1%	148; 65.5%	1.313; 89.3%	
Suicide attempt in the past	Yes	196; 11.6%	60; 26.5%	136; 9.3%	<0.0001*
	No	1.500; 88.4%	166; 73.5%	1.334; 90.7%	

* test χ^2^.

### Analysis of factors increasing the likelihood of development of anorexia nervosa among schoolchildren

Multi-factor analysis of logistic regression allowed to isolate factors that increase the likelihood of development of anorexia nervosa among the students studied.

It has been shown that independent factors significantly increasing the chance of anorexia nervosa were: sex, chronic disease, mother’s education, declaration of auto-aggression, occurrence of suicidal thoughts, high levels of school stress and physical activity. The chances of developing anorexia nervosa were highest – 7.1 times higher – for women compared to men (OR = 7.145). The use of auto-aggressive behavior increased this chance 2.8 times compared to non-auto-aggression students (OR = 2.804), and declaring suicidal thoughts increased the chance of developing mental anxiety 2.3 times (OR = 2.309) compared with students who did not report suicidal thinking. At least good physical activity increased the chance 3 times for those with lower physical activity (OR = 3.059). A mother’s higher education increased the risk of developing anorexia nervosa 5.4 times (OR = 5.436), a woman’s average education increases the risk 3.5 times (OR = 3.496) and vocational education 2.9 times (OR = 2.931) compared to primary education. The exact data are presented in Table 7.

**Table 7:** Factors influencing the development of anorexia nervosa (based on a multi-factor logistic regression model) (source: own study).

Dependent variable – eating disorders	Factor – predictor	OR** (95% CI)	*P* value
Development of anorexia nervosa	Gender (woman/man*)	7.145 (5.24–19.2)	<0.0001
	Chronic disease (yes/no*)	1.599 (1.05–2.43)	0.029
	Mother’s education (higher/primary*) (secondary/primary*) (vocational/primary*)	5.44 (1.24–23.80) 3.496 (0.79–15.39) 2.931 (0.6–13.10)	0.024 0.098 0.16
	Auto-aggression (yes/no*)	2.804 (1.93–4.8)	<0.0001
	Suicidal thoughts (yes/no*)	2.309 (1.51–3.53)	0.0001
	Stress at school >90 percentyl (yes/no*)	1.466 (1.25–1.72)	<0.0001
	At least good physical activity (≥30 min) (yes/no*)	3.059 (1.49–4.93)	<0.0001

* Reference group (OR = 1.0). ** OR standardized for variables included in the model.

## Discussion

Our study showed that the problem of eating disorders constitutes a significant health problem among Polish teenagers—the diagnostic criteria for anorexia nervosa were met by 13.3% of respondents and they were more frequently affected by girls than boys.

The first studies describing the problem of this phenomenon in Poland were conducted in 1997 by Namysłowska (1997). Recognizing then the current scientific trends and the view that eating disorders do not occur in the group of boys and men, the study was carried out only with the participation of girls. It states that anorexia nervosa occurs in 0.8–1.8% of women under 18 years of age. Similar results were obtained in studies conducted by Józefik (1999). They showed that the prevalence of anorexia nervosa in the group of Polish adolescents is in the range of 0.5–1,0%. More recent epidemiological reports (from 2011) indicate an increase in the incidence of eating disorders in Poland (Wojtyła et al., 2011; Takács et al., 2021). Wojtyła et al. (2011) indicate that anorexia nervosa occurs in approximately 0.1–5.7% of adolescents. The same paper also showed that these disorders are more common among girls than among boys (Wojtyła et al., 2011).

Such significant differences between the epidemiology of specific eating disorders in the quoted bibliographic sources and in the work of one’s own may be due to the dynamically changing living conditions and the profile of occurring diseases. It can be suspected that the group participating in the own study presents different styles of functioning and dealing with difficulties than the respondents in the Namysłowska (1997), Józefik (1999), and Wojtyła et al. (2011). Takács et al. (2021) argue that generational differences are observed in all aspects of life, including psychological resources. It has been shown that contemporary teenagers (generation Z), compared to peers from generations X and Y, are characterized by increased dysfunctions in self-regulation, less cognitive flexibility and less resistance to external events (Takács et al., 2021). Exposure to additional stress exceeds the adaptive capabilities of the body of modern youth and leads to the occurrence of non-adaptive methods of coping. Recent studies (Robinson et al., 2022; McGorry et al., 2022; Appolinario et al., 2022; Silén et al., 2020; Mitchison et al., 2020; Flatt, 2020) indicate an increasing problem of the development of mental disorders and illnesses, especially in the adolescent age group. It turns out that in the last decade there has been a critical increase in the number and severity of negative stressors with a reduction in self-help resources, which results in a deteriorating state of public mental health and leads to an increased incidence of mental disorders, including eating disorder (Wojtyła et al., 2011; Takács et al., 2021; Palacio-Ortiz et al., 2020). It is important to note that the own study was conducted during the COVID-19 pandemic, which may have correlated with the appearance or exacerbation of symptoms of eating disorders, and thus a higher rate of their occurrence. During the pandemic, there was a drastic increase in the need for advice from mental health professionals, and critically high rates of youth hospitalized in hospital psychiatric units (Palacio-Ortiz et al., 2020). It has been proven that experiences associated with COVID-19 weaken adaptive abilities and lead to increased mental problems.

Another factor explaining the differences in the incidence of eating disorders in own studies and the studies of other authors is a separate diagnostic criterion used to evaluate the individual disturbances. Eating disorders are one of the groups of diseases that have undergone significant modifications in ICD-11 compared to ICD-10 (Mandelli et al., 2019). The new version of the International Classification of Diseases and Health Problems (ICD-11) presents a different nosology of eating disorders and their subtypes.

A multi-factor analysis of logistic regression showed that female sex, mother’s education, physical activity, and the presence of auto-aggressive behavior and suicidal thoughts increased the risk of developing anorexia nervosa to the greatest extent. The findings of the current study align closely with those reported in references (Mehler et al., 2022; Broomfield et al., 2021; Pettersson et al., 2021; Pauli et al., 2022; Panero et al., 2021; West et al., 2021; Kaye and Bulik, 2021; Monteleone et al., 2019; Sagiv and Gvion, 2020; Schaumberg et al., 2021; Hartmann et al., 2019), with the exception that some authors also list traumatic sexual experiences, the absence of the father and the presence of personality disorders among these factors.

The report published by World Health Organization (2018) points out that school and family difficulties are also strongly correlated with auto-aggressive and self-destructive behavior among children and adolescents. The authors of the report indicated that being persecuted by peers or close family members, lack of close friends, physical or psychological violence and high levels of school stress are important factors in suicidal thoughts and behavior (World Health Organization, 2018).

In Półtorak (2018) conducted among Polish students, more than 60% of respondents admitted that they experience stress in school. This stress occurs in a similar group of respondents, regardless of the level of education (primary and secondary school), but is significantly more common in girls than boys (Półtorak, 2018). The highest levels of stress affect as much as 22% of elementary school students and 10% of primary school pupils. 52.3% of students anxiety, 23.4% felt sad when they went to school, and 28.8% experienced periodic panic attacks (Półtorak, 2018; Wosik-Kawala, 2019). Similar results were obtained in Wosik-Kawala (2019), in which 17.4% of students admitted that they did not go to school with pleasure. These results seem to be identical to those obtained in self-employment, where 75% of respondents admitted that school life is more stressful than family life. The maximum sense of stress (>90 percentile) occurred in as much as 9.4% of respondents.

The highest level of family stress was associated with 10.0% of all respondents and 18.6% of those with a possible diagnosis of anorexia nervosa. Specialists in the provision of assistance to victims of domestic violence must be prepared for appropriate assistance. The pandemic reality has radically changed the functioning of some families, including increasing victimization rates both during and after the pandemics. The effects of violence are never a summary response to the injuries suffered, but they exhibit a negative synergistic effect by increasing the risk of many mental illnesses and disorders, including eating disorder. New challenges must therefore be addressed and innovative solutions must be sought based on a family-centric approach (Pereda and Díaz-Faes, 2020).

The use and abuse of psychoactive substances is a significant aspect in the context of anorexia nervosa, highlighting the complexity of the psychopathological mechanisms underlying this disorder (Monteleone et al., 2019; Sagiv and Gvion, 2020; Devoe et al., 2021). Individuals suffering from anorexia nervosa often resort to substances such as alcohol, nicotine, marijuana, laxatives, and stimulants—including amphetamines or caffeine—as a means of suppressing appetite, reducing anxiety, and improving mood (Devoe et al., 2021; Ramsewak et al., 2022; Dolcini, 2024). In their case, psychoactive substances serve a compensatory function by alleviating emotional tension, school or family-related stress, and depressive symptoms. Importantly, anorexia nervosa and substance use disorders frequently share the same risk factors, such as childhood trauma, impulsivity, anxiety disorders, and dysfunctional family relationships (Monteleone et al., 2019; Sagiv and Gvion, 2020). Research shows that adolescents diagnosed with anorexia nervosa are significantly more likely than their healthy peers to report using psychoactive substances, which considerably worsens the course of the illness and increases the risk of hospitalization, self-harming behaviors, and even death (Palacio-Ortiz et al., 2020; Monteleone et al., 2019; Sagiv and Gvion, 2020; Pereda and Díaz-Faes, 2020; Devoe et al., 2021; Ramsewak et al., 2022; Dolcini, 2024). Abuse of such chemicals can lead to serious somatic complications, metabolic disorders, and the exacerbation of mental health conditions, making treatment more complex and less effective (Pereda and Díaz-Faes, 2020; Devoe et al., 2021; Ramsewak et al., 2022; Dolcini, 2024). In our study, as many as 60.2% of participants who met the diagnostic criteria for anorexia nervosa reported psychoactive substance abuse. This result indicates that access to psychoactive substances is widespread and represents a serious problem. Therefore, in the case of an anorexia nervosa diagnosis, it is essential to consider the risk of co-occurring addictions and to implement a multidisciplinary therapeutic approach that combines psychiatric, psychological, and nutritional support, along with community-based interventions.

In conclusion, the study revealed that 13.3% of respondents met the diagnostic criteria for anorexia nervosa. Individuals with a possible diagnosis of this disorder experienced significantly higher levels of both school and family stress compared to those who did not meet the criteria. Additionally, psychoactive substance abuse was found to be a prevalent issue among those with a potential diagnosis of anorexia nervosa. Furthermore, self-destructive and auto-aggressive behaviors were notably more frequent in this group, highlighting the complex interplay of psychological distress and harmful coping mechanisms associated with the disorder.

## Data Availability

The raw data supporting the conclusions of this article will be made available by the authors, without undue reservation.
